# SLC26A9 in airways and intestine: secretion or absorption?

**DOI:** 10.1080/19336950.2023.2186434

**Published:** 2023-03-03

**Authors:** Karl Kunzelmann, Raquel Centeio, Jiraporn Ousingsawat, Khaoula Talbi, Ursula Seidler, Rainer Schreiber

**Affiliations:** a Institut für Physiologie, Universität, Universitätsstraße 31, Regensburg, Germany; b Department of Gastroenterology, Hannover Medical School, Hannover, Germany

**Keywords:** Anoctamin, SLC26A9, epithelial transport, cystic fibrosis, asthma, inflammatory airway disease

## Abstract

SLC26A9 is one out of 11 proteins that belong to the SLC26A family of anion transporters. Apart from expression in the gastrointestinal tract, SLC26A9 is also found in the respiratory system, in male tissues and in the skin. SLC26A9 has gained attention because of its modifier role in the gastrointestinal manifestation of cystic fibrosis (CF). SLC26A9 appears to have an impact on the extent of intestinal obstruction caused by meconium ileus. SLC26A9 supports duodenal bicarbonate secretion, but was assumed to provide a basal Cl- secretory pathway in airways. However, recent results show that basal airway Cl- secretion is due to cystic fibrosis conductance regulator (CFTR), while SLC26A9 may rather secrete HCO_3_-, thereby maintaining proper airway surface liquid (ASL) pH. Moreover, SLC26A9 does not secrete but probably supports reabsorption of fluid particularly in the alveolar space, which explains early death by neonatal distress in Slc26a9-knockout animals. While the novel SLC26A9 inhibitor S9-A13 helped to unmask the role of SLC26A9 in the airways, it also provided evidence for an additional role in acid secretion by gastric parietal cells. Here we discuss recent data on the function of SLC26A9 in airways and gut, and how S9-A13 may be useful in unraveling the physiological role of SLC26A9.

## Introduction

The mammalian SLC26 family comprises 11 transport proteins with different physiological functions and variable expression in the human body. SLC26 proteins exchange anions and function as electroneutral or electrogenic transporters. A number of diseases are connected to mutations in SLC26 genes, like chondrodysplasia (SLC26A2), congenital chloride-losing diarrhea (SLC26A3), or Pendred Syndrome (SLC26A4) [1]. The underlying disease-causing mutations induce folding defects, resulting in impaired trafficking of these transporters from the endoplasmic reticulum to the cell surface. Alternatively, mutations may directly affect transport functions when localized within the anion-binding site [2,3]. SLC26A9 has not been directly linked to disease, but sequencing data identified variants of SLC26A9 that are associated with an increased incidence of meconium ileus or diabetes observed in patients with cystic fibrosis (CF) [4–9]. While there is good evidence for the role of SLC26A9 in gastrointestinal transport, it remains unclear whether it affects CF lung disease severity and airway response to CFTR-therapeutics [10,11]. Currently available data on tissue expression and biophysical properties of SLC26A9 provide an incomplete and somewhat confusing picture of the physiological function of SLC26A9 that can operate as an uncoupled chloride transporter [12].

## Expression of SLC26A9 in the lung and its potential role for generation of airway surface liquid

SLC26A9 was shown to be located at the luminal side of lung bronchiolar and alveolar epithelium [13]. Patch clamp studies identified whole cell currents associated with overexpression of SLC26A9, and suggested a function of SLC26A9 as Cl- channel rather than anion exchanger [14,15]. Thus SLC26A9 was proposed to contribute to hydration of the airway surface liquid (ASL), in addition to cAMP-activated CFTR [14–18]. The use of the CFTR-inhibitor GlyH101 to inhibit SLC26A9 in airway epithelial cells in order to dissect out the contribution of SLC26A9 to Cl- transport is difficult. That’s because GlyH101 is a potent inhibitor of CFTR, and also shows off-target effects by inhibiting Ca^2+^ channels [19,20]. As outlined below, there is now good evidence that CFTR, but not SLC26A9 generates a basal airway Cl- conductance even in the absence of any stimuli [21].

There is a large body of evidence that CFTR requires increase in intracellular Ca^2+^ to be fully activated [22–25]. CFTR activity is largely supported by the Cl- channel TMEM16A (ANO1). This is explained by the fact that TMEM16A, apart from being a Cl- channel, also augments intracellular Ca^2+^ signals elicited by activation of phospholipase-coupled receptors [26–28]. Due to its effect on intracellular Ca^2+^, TMEM16A also supports trafficking of CFTR to the apical epithelial membrane [20,29–31]. Remarkably, patients homozygous for a loss of function mutation in TMEM16A and TMEM16A-knockout mice loose CFTR function [20,32].

## Basal airway Cl- secretion by CFTR

**Figure 1.**
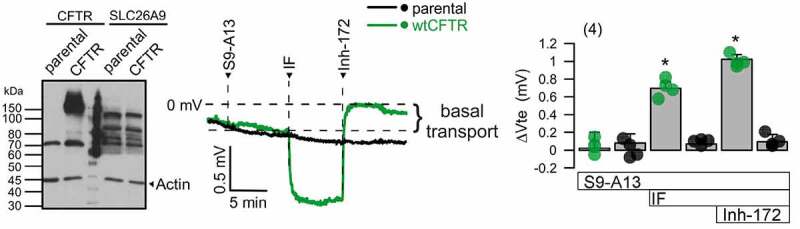
*CFTR but not SLC26A9 does contribute to basal Cl*- *transport in CFBE human airway epithelial cells*. Left: Western blot of CFTR in CFBE-wtCFTR human airway epithelial cells, but not in CFBE-parental cells lacking expression of CFTR (parental). In contrast, endogenous SLC26A9 is expressed in both CFBE-wtCFTR and in CFBE-parental cells. The four bands detected are due to expression of the different splice variants of SLC26A9. Middle: Original Ussing chamber recordings obtained from CFBE-parental and CFBE-wtCFTR cells under open circuit conditions. The basal transepithelial voltage (Vte) reflecting the basal transport in nonstimulated CFBE cells was not affected by S9-A13 (5 µM), suggesting that SLC26A9 does not contribute to basal Cl- secretion in airways. In CFBE-wtCFTR cells but not in CFBE-parental cells, IBMX and forskolin (IF; 100/2 µM), induced a negative voltage deflection due to activation of CFTR-dependent Cl- secretion. The CFTR inhibitor 172 (Inh-172) inhibited the IF-activated transport and also the basal transport, suggesting that CFTR is in charge of both basal and stimulated transport. Right: Summary of the changes in transepithelial voltage due to different manoevers. Cell culture and Ussing chamber techniques have been reported in previous publication [15]. Mean ± SEM (number of experiments). *indicate significant effects of if and inh-172 (*p* < 0.05; ANOVA).

Larsen et al showed that both CFTR and SLC26A9 are expressed in human bronchial epithelium (HBE) and that constitutive (basal) chloride channel activity is unaffected by knockdown of CFTR. They concluded that SLC26A9 is the primary source of constitutive anion secretion across HBE [18]. We could not reproduce their results in our recent study using CFTR inhibitors and the novel SLC26A9-inhibitor S9-A13 [21]. S9-A13 potently inhibits whole cell currents and iodide quenching in SLC26A9-overexpressing cells, but does not inhibit CFTR. In mouse trachea or in highly differentiated human airway epithelial cells (which both express SLC26A9 and CFTR), S9-A13 had no effect on either basal or cAMP-activated Cl- secretion, indicating that SLC26A9 is not in charge of airway Cl- secretion [21]. In contrast CFTRinh172 completely inhibited basal and cAMP-induced transport. Also in CFBE human airway epithelial cells, S9-A13 had no effect on transepithelial voltage, while CFTRinh172 inhibited basal (and activated) Cl- secretion (Figure 1). These data are in perfect agreement with earlier work, demonstrating basal airway Cl- secretion by CFTR [33–36].

The cause for this basal CFTR activity in the absence of an increase in intracellular cAMP remains obscure. Basal CFTR activity was maintained even after inhibition of endogenous adenylate cyclase type 1 or after blocking adenosine 2AB-receptors. This makes it unlikely that luminal purinergic signaling indirectly activates CFTR by increasing intracellular cAMP [21,37]. For other members of the SLC26 family, like SLC26A4, it was shown to enhance CFTR-dependent secretion [38]. It might be possible that also SLC26A9 upregulates CFTR-activity. Such a SLC26A9/CFTR cooperativity probably requires a polarized environment, as we could not detect it in overexpressing HEK293 cells [15]. A possible activation of CFTR by SLC26A9 should be examined in airway tissues. Here, the novel SLC26A9 inhibitor S9-A13 may be useful [21].

## Airway HCO_3_- secretion

**Figure 2.**
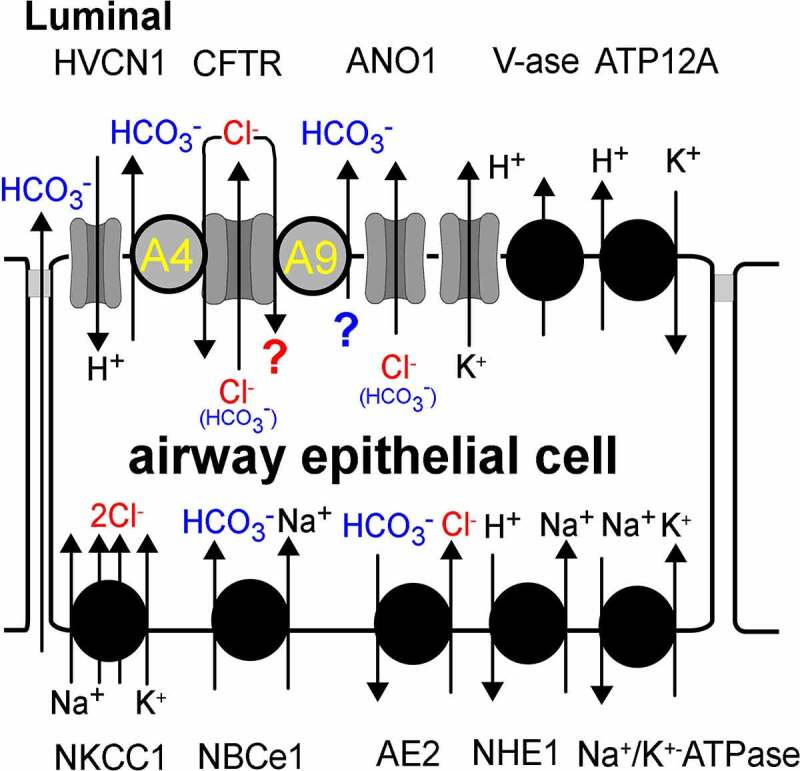
*Model for HCO*_*3*_- *and Cl*- *transport in airway epithelial cells*. Ion transport proteins are known to be expressed in the luminal and basolateral membrane of airway epithelial cells: HVCN1, voltage-gated hydrogen channel 1; A4, SLC26A4 (Pendrin); A9, SLC26A9; ANO1, Ca^2+^ activated Cl- channel anoctamin 1 (TMEM16A); V-ase, vacuolar H±ATPase; ATP12A, H+/K+ - ATPase; NKCC1, Na+/K+/2Cl- -cotransporter; NBCe1, electrogenic Na+/HCO_3_- cotransporter 1; AE2. anion exchanger type 2; NHE1, Na+/H+-exchanger 1. An apical Cl-/HCO_3_- exchange by SLC26A9 requires more experimentation.

While CFTR maintains basal Cl- flux to the luminal side of the airway epithelium, our recent findings give a first hint as to the possible role of SLC26A9 in HCO_3_- secretion under basal (non-stimulated) conditions [21]. Numerous transport proteins are located in luminal and basolateral membranes and contribute to airway HCO_3_- transport, which makes it difficult to dissect out the contribution of each individual channel, transporter or pump [21,39] (Figure 2). Obviously, an essential portion of HCO_3_- is secreted by CFTR, although its permeability for HCO_3_- (P_Cl-_/P_HCO3-_~4) is limited and at the same time the abundance of Cl- ions ([Cl-]/[HCO_3_-] ~ 4) is much higher. Determination of ASL pH is technically demanding and expression levels and location of bicarbonate transporters vary significantly between airways of different species. Nevertheless, an overall picture emerged connecting lack of CFTR with airway acidification, mucus abnormalities, and attenuation of airway defense, finally leading to a CF lung phenotype [40–43]. It should be mentioned however that some studies did not detect an acidic ASL pH in children with CF or in airways of CFTR knockout piglets [36,44]. We may speculate that these CFTR-/- piglets showed normal ASL pH because SLC26A9 expression in the apical membrane of airway epithelial cells from CFTR-/- piglets is unaffected {Ousingsawat, 2022 #9480}.

The contribution of additional apical HCO_3_- transporters is discussed for years. SLC26A4 (Pendrin) has now been shown to secrete substantial amounts HCO_3_-, particularly when upregulated by interleukin 4 or 13 [38,45]. In renal intercalated type B (IC-B) cells, we demonstrated regulation of Pendrin activity by CFTR. Lack of expression or dysfunctional CFTR in IC-B cells leads to loss of HCO_3_- secretion in the collecting duct which explains metabolic alkalosis observed in people with cystic fibrosis [46,47]. Although the detailed molecular mechanisms are still missing, the data suggest that CFTR provides luminal Cl- to be recycled by Pendrin, which in turn secretes HCO_3_- (Figure 2). Thus, Pendrin could operate together with CFTR according to the classical carrier/channel recycling concept, described by Novak and Greger [48]. Activation of CFTR by cAMP/PKA further enhances the activity of Pendrin, while Pendrin enhances CFTR-mediated Cl- secretion. Whether CFTR stimulates Pendrin via molecular interaction or simply by providing luminal Cl- to drive HCO_3_- transport, is currently unclear. A STAS (SLC26)/R-domain (CFTR) interaction has been described for pancreatic SLC26A3 and SLC26A6, which is likely to be involved also in regulation of SLC26A4 activity by CFTR [49,50]. The elegant model developed by Muallem and coworkers provides a concept for coordinated transport of HCO_3_- and Cl-, matched by the activity of basolateral and apical anion transport. This model includes a shift of CFTR – mediated Cl- to HCO_3_- transport. This shift occurs in the distal part of the pancreatic duct through low intracellular [Cl-] that activates the Cl- sensitive kinase WNK1 (with-no-lysine kinase), OSR1 (oxidative stress-responsive kinase 1) and SPAK (sterile 20/SPS1-related proline/alanine-rich kinase) [51].

## Neonatal distress due to defective Cl- absorption in SLC26A9 knockout mice

If SLC26A9 truly secretes HCO_3_- in exchange with Cl- ions, it may even serve in fluid reabsorption rather than secretion (Figure 2). Homozygous Slc26a9 knockout mice typically die within 24 h after birth [52,53] and only a few animals survive. Animals that overcome the critical postnatal phase do not present a pulmonary phenotype [54]. Alveolar type (AT)I and ATII cells maintain normal alveolar fluid homeostasis. Functional defects in either ATI or ATII may result in acute lung injury and acute respiratory distress syndrome [55]. Slc26a9 is expressed in the lungs most prominently in ATII cells. An early abstract by the Seidler team already suggested a defect in airway liquid absorption rather than a lack of Cl- secretion that leads to early neonatal death of SLC26A9 knockout animals [52]. A more recent abstract reports little airspace in the lungs of Slc26a9 knockout animals, which supports this concept [53]. It will be interesting to learn whether indeed alveolar fluid absorption is reduced in the absence of Slc26a9, and whether SLC26A9-mediated fluid absorption is also observed in the bronchial epithelium. Hypothetically, inhibition of SLC26A9 could even improve CF lung disease by shifting airway transport toward fluid secretion and increase in ASL height. This could be nicely examined *in vitro*.

## Apical expression of SLC26A9 in airways

Apart from Pendrin, also SLC26A9 is expressed in airway epithelial cells. Using immunohistochemistry, we demonstrated location of SLC26A9 in the apical membrane of ciliated epithelial cells of human, mouse, and piglet airways, as well as ciliated Bci-NS1 and CFBE airway epithelial cells [21,56]. In contrast, SLC26A9 was not detected in airways of CF-patients homozygous for F508del-CFTR. As pointed out above, airways of piglets lacking expression of CFTR (CFTR-/-) showed normal apical localization of SLC26A9, comparable to wild type animals. This suggests normal trafficking of SLC26A9 to the apical membrane in the absence of CFTR, while F508del-CFTR inhibits expression of SLC26A9 in the apical membrane [56]. In contrast to superficial airways, SLC26A9 was not expressed in submucosal glands, which makes sense since submucosal glands are the true place of secretion. The clear apical staining of SLC26A9 is surprising as single cell mRNA analysis demonstrated relatively low expression of SLC26A9 in ciliated cells, when compared to alveolar ATII cells (https://www.proteinatlas.org/ENSG00000174502-SLC26A9/single+cell+type). It is currently not known whether these transcript data correspond to protein expression. SLC26A9 is clearly labeled in airways from individuals expressing wtCFTR, and no artefactual staining of cilia was detected. Most important, tissues expressing F508del-CFTR stained negative for apical SLC26A9. Nevertheless, there is always a possibility for false positive stainings.

**Figure 3.**
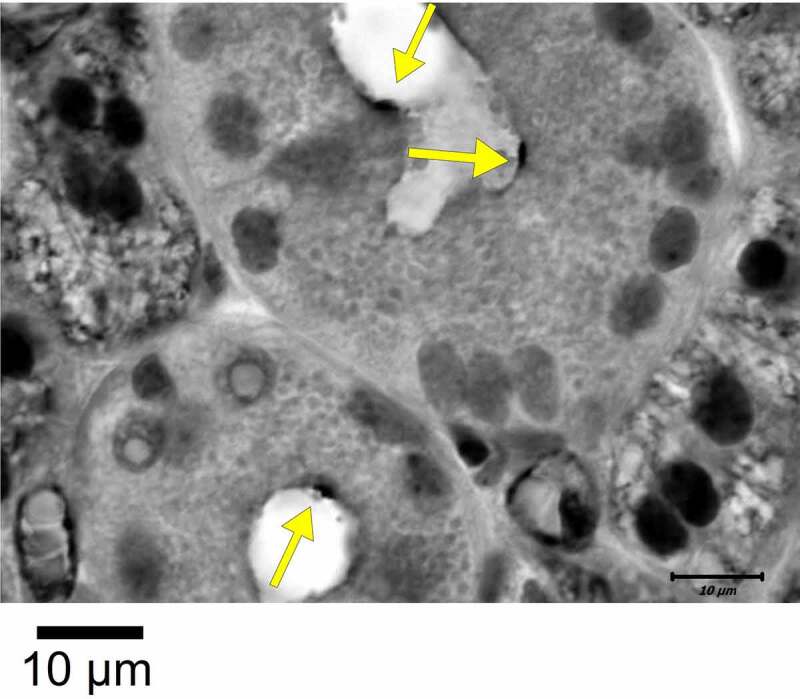
*CFTR hyperexpressing cells (ionocytes; yellow arrows) in submucosal glands*. Bar = 10 µm. Technical details are described in [21]. .

Along this line, a nonspecific binding was detected for anti-CFTR antibodies in ciliated cells and single cell RNA analysis (scRNAseq) now suggests predominant expression of CFTR in ionocytes and secretory cells [57–60]. It should be mentioned that already 2 decades ago, a low and variable CFTR-mRNA expression was found in superficial airway epithelial cells, which nevertheless produce clearly detectable CFTR currents [61–63]. Engelhardt and coworkers and our own team detected CFTR hyperexpressing cells (probably ionocytes) predominantly in submucosal glands, a finding that was confirmed by many other laboratories [64–69] (Figure 3).

## HCO_3_- transport by SLC26A9

**Figure 4.**
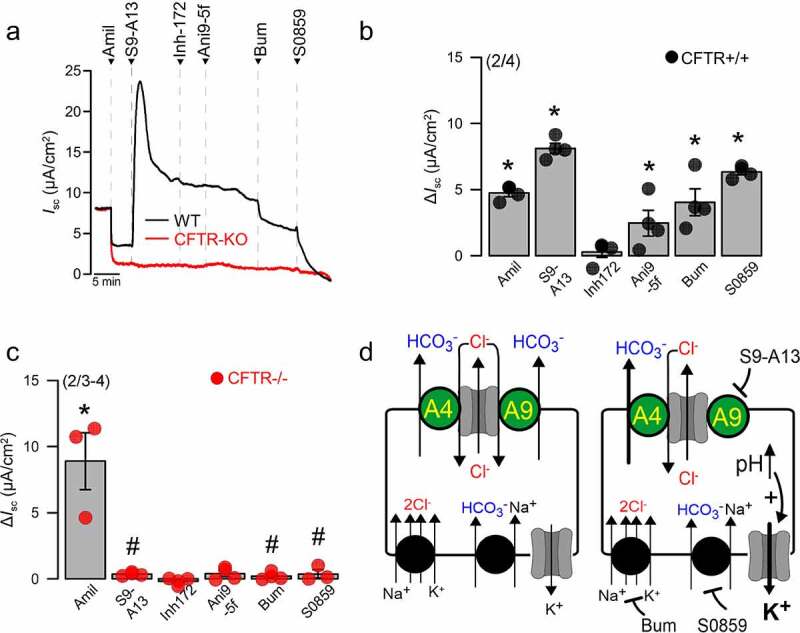
*SLC26A9-inhibitor S9-A13 possibly activates K*^*+*^ *currents in primary porcine airways through cytosolic alkalization*. A) Short circuit currents in primary porcine airways from CFTR+/+ (WT) and CFTR-/- (CFTR-KO) piglets. Activation of a partially transient short circuit current (Isc) in WT but not CFTR-KO airways, by application of S9-A13 (5 µM). Isc was not inhibited by the CFTR inhibitor CFTRinh172 (Inh-172; 5 µM, luminal) or the TMEM16A inhibitor Ani9-5F (5 µM, luminal), but was completely blocked by the NKCC1-inhibitor bumetanide (Bum; 100 µM, basolateral) and inhibition of NBCe1 by S0859 (30 µM, basolateral). Note the enhanced amiloride (10 µM) -sensitive Isc in CFTR-KO airways. B,C) Summary of the effects induced by the different inhibitors in WT and CFTR-KO airways. D) Hypothetical transport model explaining the activation of Isc by S9-A13, which is inhibited by Bum/S0859. Primary cell culture of CFTR+/+ and CFTR-/- piglet airway cells and short circuit measurements have been described in a previous publication [36]. Mean ± SEM (number of experiments). *indicates significant effect of inhibitor (P < 0.05; paired t-test) #indicates significant difference for the effects of S9-A13, bumetanide, and S0859 in CFTR-/- airways (*p* < 0.05; ANOVA).

Our recent data demonstrate acidification of the ASL pH induced by the SLC26A9 inhibitor S9-A13 [21]. These data have been obtained under thin film conditions in highly differentiated human airway epithelial cells. As mentioned above, we found equal expression of SLC26A9 in airways from CFTR+/+ and CFTR-/- knockout piglets [56], indicating expression of SLC26A9 also in the absence of CFTR. Interestingly, short circuit currents (Isc) were activated in CFTR+/+ but not CFTR-/- primary piglet airway epithelial cells by S9-A13 (Figure 4). The partially transient Isc activated by S9-A13 was not blocked by CFTRinh172 or Ani9-5F, suggesting no contribution of CFTR or TMEM16A. However, inhibition of the Na+/2Cl-/K+-cotransporter NKCC1 by bumetanide and inhibition of the Na+/HCO_3_--cotransporter NBCe1 by S0859 completely abolished S9-A13 induced transport. None of this was observed in the absence of CFTR (CFTR-/-). While the underlying mechanisms still remain obscure, we speculate that in the presence of CFTR, SLC26A9 can operate as a transporter for HCO_3_-. Accordingly, acute inhibition of SLC26A9 would inhibit HCO_3_- secretion and cause intracellular alkalosis, which may activate pH-sensitive K+ channels [70] and possibly enhance secretion of HCO_3_- via Pendrin (Figure 4).

Several previous studies reported HCO_3_- transport by SLC26A9 [13,71–75]. However, other teams did not find evidence for a HCO_3_- permeability of SLC26A9 [12,14,16,17]. Our own pH measurements in SLC26A9-overexpressing HEK293 cells did also not show HCO_3_- transport [21]. We hypothesize that HCO_3_- permeability of SLC26A9 may require co-localization with CFTR in the apical membrane of polarized epithelia. A recent study on perfused mouse bronchioles supports the concept of HCO_3_- secretion by SLC26A9 in the airway epithelium [76]. This, however, needs further investigation.

## Upregulation of CFTR and basal airway Cl- transport by IL-4

Adult SLC26A9-/- mice do not show a pulmonary phenotype and basal airway transport assessed in Ussing chamber experiments appeared undisturbed [54]. However, after induction of an asthma-like phenotype by intratracheal application of IL-13, wild type animals showed enhanced basal airway transport, while in SLC26A9-/- animals basal equivalent short circuit currents were attenuated. This was correlated to apparent airway mucus plugging observed in the knockout animals. Our recent study also suggested larger basal transport in CFBE airway epithelial cells after IL-13 treatment, which enhanced membrane expression of SLC26A9 [56]. Again, this was not observed in CFBE cells expressing F508del-CFTR. Notably, IL-13 also strongly upregulates expression of CFTR and TMEM16A [30]. Thus, enhanced basal ion transport after exposure to IL-13 is likely due to upregulation of basal CFTR/TMEM16A conductance.

## Gastrointestinal function of SLC26A9

**Figure 5.**
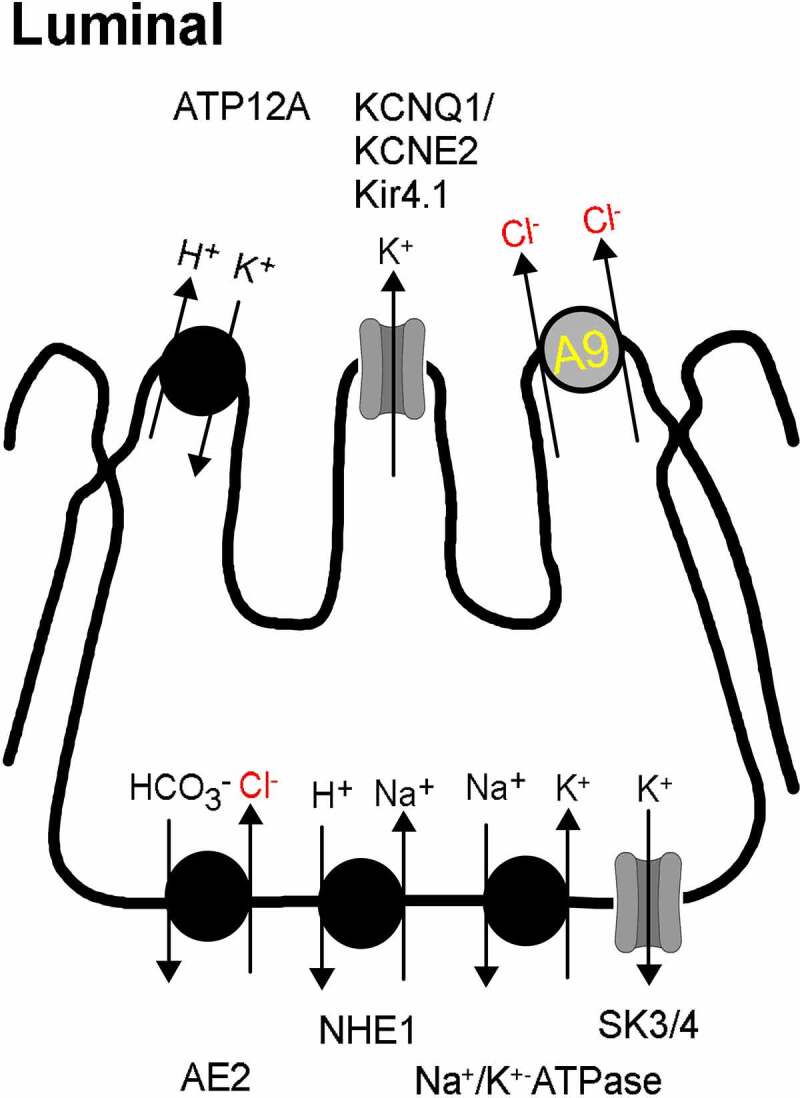
*Model for H*^*+*^ *secretion by gastric parietal cells*. Ion transport proteins in the luminal and basolateral membrane in acid secreting parietal cells. A9, SLC26A9; ATP12A, H+/K+ - ATPase; KCNQ1/KCNE2, potassium voltage-gated channel subfamily Q member 1 (K_v_7.1, KvLQT1)/potassium voltage-gated channel subfamily E member 2 (KCNE2; mink-related peptide 1); Kir4.1, ATP-sensitive inward rectifier potassium channel 10 (*KCNJ10);* AE2, anion exchanger type 2; NHE1, Na+/H+-exchanger 1; SK3/4, small conductance calcium-activated potassium channel 3 (K_Ca_2.3; KCNN2)/intermediate conductance calcium activated potassium channel (K_Ca_3.1, KCNN4).

**Figure 6.**
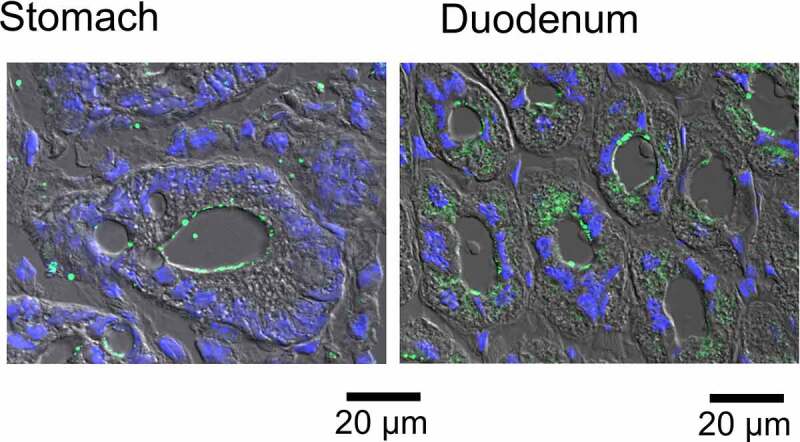
*Apical localization of SLC26A9 in gastric parietal cells and duodenal crypts of Lieberkühn*. Green, SLC26A9; blue, DAPI. Bars = 20 µm. Technical details are described in [21]. .

SLC26A9 is highly expressed in stomach, less in duodenum, and not detected in the distal GI tract. Early work suggested that SLC26A9 expressed in surface epithelial cells of mouse stomach, mediates bicarbonate secretion, and protects the gastric mucosa [72,77]. A role of SLC26A9 for HCO_3_- secretion was also found in the proximal duodenum, which shows pronounced expression of CFTR, in contrast to low levels of CFTR in the stomach [75,78]. Acid secreting gastric parietal cells require an apical Cl- conductance. In the past, a number of different Cl- channels have been proposed to form the apical channel in parietal cells, including ClC-2 [79,80], CFTR [81], or intracellular Cl- channels like Clic6 and parchorin [82]. An earlier study demonstrated reduced gastric acid secretion in SLC26A9 knockout mice. The study suggested a role of SLC26A9 for H+ secretion by providing a Cl- secretory pathway to balance the H+ pump activity [73]. It should be considered, however, that deletion of Slc26a9 also caused a loss of tubulovesicles in parietal cells. It is therefore not clear whether structural abnormalities were the cause for reduced H+ secretion or a true loss of Cl- conductance in parietal cells [73]. Nevertheless, in our recent study the SLC26A9 inhibitor S9-A13 inhibited histamine-induced H+ secretion, suggesting a role of SLC26A9 for acid secretion by providing a Cl- secretory pathway [21] (Figure 5). As no CFTR expression was found in these cells, we hypothesized that SLC26A9 can operate as a Cl- transporter in the absence of CFTR. Clearly, the role of SLC26A9 for H+ and HCO_3_- secretion in the GI-tract should be further investigated, ideally in inducible tissue-specific Slc26a9-knockout animals. In these studies, the inhibitor S9-A13 could be useful. Moreover, improved immunohistochemistry of SLC26A9 in the stomach is required, which allows a more precise cellular localization of SLC26A9, under both resting conditions and after stimulation with secretagogues (Figure 6).

## SLC26A9 – operating in different modes?

In our recent report we propose that SLC26A9 may operate differentially, depending on coexpression of CFTR [21]. In fact, an earlier report discussed three different modes of operation for SLC26A9, as *n*Cl- -HCO_3_- exchanger, Cl- channel, and Na+-anion cotransporter, enabled by a tissue-speciﬁc regulation [71]. CFTR could indeed live up to its name by controlling the mode of action of SLC26A9. A number of papers already demonstrated physical and functional interaction of CFTR with various members of the SLC26A solute transporter family (SLC26A3,4,6,8,9). Molecular interaction probably takes place through CFTR’s R domain and the STAS (Sulfate Transporter and AntiSigma factor antagonist) domain of the SLC26A proteins [1,14,46,49–51,83–85]. The novel inhibitor S9-A13 may help to shed more light into this functional relationship.
